# Non-alcoholic fatty liver disease risk prediction model and health management strategies for older Chinese adults: a cross-sectional study

**DOI:** 10.1186/s12944-023-01966-1

**Published:** 2023-11-25

**Authors:** Hong Pan, Baocheng Liu, Xin Luo, Xinxin Shen, Jijia Sun, An Zhang

**Affiliations:** 1 Department of Health Management, School of Public Health, Shanghai University of Traditional Chinese Medicine, Shanghai, China; 2 Shanghai Collaborative Innovation Centre of Health Service in Traditional Chinese Medicine, Shanghai University of Traditional Chinese Medicine, Shanghai, China; 3 School of Public Health, Shandong First Medical University, Shandong, China; 4 Department of Mathematics and Physics, School of Pharmacy, Shanghai University of Traditional Chinese Medicine, Shanghai, China

**Keywords:** Non-alcoholic fatty liver disease, Nomogram, Bayesian network, Chinese older adults, Health management strategies

## Abstract

**Background:**

Non-alcoholic fatty liver disease (NAFLD) is a common chronic liver condition that affects a quarter of the global adult population. To date, only a few NAFLD risk prediction models have been developed for Chinese older adults aged ≥ 60 years. This study presented the development of a risk prediction model for NAFLD in Chinese individuals aged ≥ 60 years and proposed personalised health interventions based on key risk factors to reduce NAFLD incidence among the population.

**Methods:**

A cross-sectional survey was carried out among 9,041 community residents in Shanghai. Three NAFLD risk prediction models (I, II, and III) were constructed using multivariate logistic regression analysis based on the least absolute shrinkage and selection operator regression analysis, and random forest model to select individual characteristics, respectively. To determine the optimal model, the three models’ discrimination, calibration, clinical application, and prediction capability were evaluated using the receiver operating characteristic (ROC) curve, calibration plot, decision curve analysis, and net reclassification index (NRI), respectively. To evaluate the optimal model’s effectiveness, the previously published NAFLD risk prediction models (Hepatic steatosis index [HSI] and ZJU index) were evaluated using the following five indicators: accuracy, precision, recall, F1-score, and balanced accuracy. A dynamic nomogram was constructed for the optimal model, and a Bayesian network model for predicting NAFLD risk in older adults was visually displayed using Netica software.

**Results:**

The area under the ROC curve of Models I, II, and III in the training dataset was 0.810, 0.826, and 0.825, respectively, and that of the testing data was 0.777, 0.797, and 0.790, respectively. No significant difference was found in the accuracy or NRI between the models; therefore, Model III with the fewest variables was determined as the optimal model. Compared with the HSI and ZJU index, Model III had the highest accuracy (0.716), precision (0.808), recall (0.605), F1 score (0.692), and balanced accuracy (0.723). The risk threshold for Model III was 20%–80%. Model III included body mass index, alanine aminotransferase level, triglyceride level, and lymphocyte count.

**Conclusions:**

A dynamic nomogram and Bayesian network model were developed to identify NAFLD risk in older Chinese adults, providing personalized health management strategies and reducing NAFLD incidence.

**Supplementary Information:**

The online version contains supplementary material available at 10.1186/s12944-023-01966-1.

## Background

Affecting more than one-quarter of the world's adult population, non-alcoholic fatty liver disease (NAFLD) is a common chronic hepatopathy [1–4]. The prevalence of NAFLD is on the rise worldwide, with rates from 13 percent in Africa to 42 percent in South East Asia [5, 6]. Particularly, NAFLD prevalence in China is expected to reach 315 million by 2030 [7]. There is growing evidence that the incidence of NAFLD is significantly higher in the elderly, with prevalence rates of 40.3% and 39.2% in people aged 60–74 years and over 74 years, respectively [8–10]. In addition, several risk factors for the development of NAFLD, such as high blood pressure, hyperlipidemia, and obesity, are more prevalent in older adults, which means they are at greater risk of developing NAFLD [11]. Compared to other populations, older adults are at a higher risk of developing NAFLD, which has a significant impact on patient's quality of life and a substantial economic burden on families and healthcare systems. Meanwhile, patients with NAFLD are at an increased risk of developing cardiovascular ailments including hypertension, coronary artery disease, and arrhythmia, which contribute to an increase in clinical cardiovascular incidence rate and mortality [12, 13]. Furthermore, there will be fat accumulation in the liver of patients with NAFLD, which may affect the normal insulin signal transmission and function, leading to the development of insulin resistance (IR) and thus increasing the risk of type II diabetes mellitus [11, 14, 15]. However, no drugs have been granted regulatory approval for the treatment of NAFLD [3, 14]; therefore, lifestyle intervention is the only treatment method for NAFLD. Lifestyle intervention mainly aims to identify and manage the risk factors of the disease, thereby playing a role in alleviating and controlling the condition. The risk factors for NAFLD can interact and promote disease progression through complex mechanisms. Therefore, identifying the high-risk population and providing targeted health management interventions are crucial.

Prediction models integrate risk factors to identify susceptible individuals for a specific condition, enabling preventive interventions to be implemented [16]. Various NAFLD prediction models have been developed for European, North American, and some Asian populations [17, 18]. For example, Lee et al. constructed a Hepatic steatosis index (HSI) based on the Korean population using multivariate logisitic regression analysis, which is being widely used [19]. The Fatty liver index, developed by Bedogni G et al. based on the Italian population, is now one of the commonly used predictive models for NAFLD [20]. Furthermore, seven machine learning algorithms were used in a cross-sectional study of 15,315 Chinese individuals to develop and validate NAFLD prediction models [21]. However, this type of model was not developed using preliminary indicator screening and included many irrelevant variables, which may reduce its prediction accuracy. In addition, owing to differences in genetic and environmental (economic level, diet, lifestyle, and climate) factors, the distribution and severity of risk factors for NAFLD vary across populations, resulting in the underperformance of these prediction models when applied to other ethnic groups [22]. Several models for predicting NAFLD risk have been established for the Chinese population [18, 21, 23, 24]. Zhou et al. created a NAFLD risk nomogram for Chinese adults utilizing the Lasso logistic model [25]; whereas Liang et al. developed a precise scoring tool to detect NAFLD in individuals aged 45–70 years [26]. However, few NAFLD prediction models exist for older adults (≥ 60 years), and there is a lack of research focusing specifically on NAFLD risk prediction among older adults in China.

Therefore, based on the machine learning method, this study identified several key variables with high predictive ability in the Chinese elderly population, and used HSI and ZJU index as comparison models to establish a more suitable NAFLD risk prediction model for the Chinese older adults. In addition, Bayesian networks are used to analyze the correlation between variables to explore the correlation between key indicators and provide reference for existing disease management plans.

## Methods

### Study population

Data from 11,136 permanent community residents of Han ethnicity who participated in a screening programme for chronic disease prevention at the Zhangjiang and Beicai Community Health Services Centres in Zhangjiang Town of Shanghai’s Pudong New District between April 2016 and July 2017 were collected for this study. Using Tukey's Test to identify outliers, values less than Q1-3 interquartile range (IQR) or greater than Q3 + 3 IQR are considered outliers [27]. Since outliers in the data can cause the model to be overly sensitive or unstable, ultimately leading to reduced accuracy and reliability, it was necessary to remove the outlier sample while maintaining sufficient sample size. In addition, missing data and data from residents aged < 60 years were also excluded from this study. Therefore, the final analysis included data from 9,041 participants. The training and validation datasets included data from 3,776 and 5,265 patients collected in 2016 and 2017, respectively.

### Outcome measures

Physical examination required 12-h overnight fasting, and blood pressure and anthropometric parameters were measured the next morning. Before measurement, participants were given a minimum of 5 min to rest, and the blood pressure was recorded using an electronic sphygmomanometer (Biospace; Cheonan, South Korea).

Furthermore, to evaluate anthropometric indicators, participants were required to take off thick clothes, shoes, and hats and urinate and defecate before the examination. The steps recommended by international standards were adopted. Height and weight were determined using electronic height and weight measuring instruments (Zhengzhou Shengyuan Instrument Co., Ltd., Zhengzhou, China). Waist and hip circumferences were measured using a medical soft ruler (Pudong New Area CDC, Shanghai, China) with 0.1 cm accuracy.

A 12-h overnight fast was required for biological specimen collection. Venous blood was collected from each subject the next morning in 2 separate collections (total 5 mL), with 2.5 mL placed in a dry tube and 2.5 mL in an EDTA tube. And vortex the EDTA tube to mix the sample. Finally, they were sent to the Laboratory Department of the Zhangjiang Community Health Service Centre for analysis by an automatic biochemical analyzer (HITACHI, Japan). Liver function, kidney function, blood lipids, and blood sugar levels were evaluated.

### Clinical diagnosis

NAFLD was diagnosed based on the clinical diagnostic criteria of the guidelines for the diagnosis and treatment of NAFLD (2010 revised version) developed by the Chinese Medical Association [28]. All three of the following conditions must be met, specifically: (1) no history of alcohol consumption, or alcohol consumption equivalent to alcohol consumption < 140 g/ week for men and < 70 g/ week for women; (2) excluding other causes of hepatic steatosis, including excessive alcohol consumption, viral hepatitis, drug-induced liver disease, autoimmune liver disease, hepatolenticular degeneration, and total parenteral nutrition; and (3) significant hepatic steatosis should be suggested by imaging or histological findings.

Regarding Article 3, due to the invasive nature of liver puncture and its potential risks, including bleeding, infection, as well as puncturing other organs, a majority of participants did not undergo this procedure. Hepatic steatosis was evaluated using an abdominal ultrasound scan, which was examined by two sonographers with at least intermediate professional titles using an ultrasound machine. Hepatic steatosis was identified by the existence of at least two of the following conditions:(1) the intrahepatic duct structure was not clearly displayed; (2) the liver's near-field echo exhibited diffusion and enhancement, surpassing the kidney's echo strength, and (3) the echo from the liver's far-field attenuated gradually.

Participants in this study must be: (1) permanent residents of Zhangjiang area in Pudong, Shanghai; (2) Han Chinese without family ties; (3) all physical examination, biochemical and sample collection information must be complete and reliable; and (4) sign an informed consent. The exclusion criteria were as follows: (1) inability to cooperate or difficulty in speech expression; (2) Other causes of hepatic steatosis include excessive drinking and diagnosis of other liver diseases; or malignant tumors; and (3) weekly alcohol consumption exceeding 140 g for males and 70 g for females.

### Data modelling

The training group was used to train the parameters of each prediction model. Feature selection carried out by using the least absolute shrinkage and selection operator (LASSO) regression and random forest (RF) model to identify key variables in the prediction model. The LASSO regression analysis reduced predictive errors by constraining model parameters and decreasing regression coefficients for certain variables [29]. Additionally, variables with regression coefficients of zero were excluded from the model, and those with regression coefficients other than zero had the highest correlation with the response variable [30]. A tenfold cross-validation was conducted for the LASSO regression analysis that centralized and normalized the included variables, selected the optimal lambda value [31], and identified the most important patient characteristics that were included as variables in the prediction models [32]. The coefficients of each characteristic variable after LASSO regression analysis were visualized using a lollipop diagram. The study utilised the RF algorithm founded on recursive feature elimination, with the training model employed to ascertain the significance of variables. The RF model was initially used to train a subset of 20 variables, evaluate their importance, and assess the model’s classification accuracy through cross-validation. Subsequently, iteratively remove the least important variables and repeat the process until all variables have been evaluated. Ultimately, 20 subsets of features were obtained along with their corresponding classification accuracies, from which the optimal subset could be selected [33]. The importance ranking of each characteristic variable in RF was obtained using the varImp function in R software and was subsequently visualized.

Prediction Model I was established based on multivariate logistic regression analysis, using the variables screened by the LASSO regression analysis as independent variables to predict the incidence of NAFLD. Prediction Model II was constructed by conducting a multivariate logistic regression analysis, with the variables identified using the RF model serving as the independent variables. The Prediction Model III was developed using multiple logistic regression analysis with the common variables screened by LASSO and RF as independent variables.

### Model evaluation

To determine the optimal model, we evaluated the performance of the models based on the following four dimensions: the receiver operating characteristic (ROC), calibration plot, decision curve analysis (DCA), and net reclassification index (NRI). The ROC curve is a tool used to visualise the discriminatory power of a predictive model by plotting a curve illustrating the correlation between true positive and false positive rates, which helps to assess the classification validity of the model at different thresholds [34]. Bootstrap resampling was performed 500 times, and the area under the ROC curve (AUC) was used to calculate the model’s discrimination ability. The larger the corresponding area, the stronger the corresponding disease discrimination ability. A calibration plot was utilised to conduct 100 rounds of bootstrap resampling in order to assess the precision of model fitting. DCA was a method used to evaluate and compare the effectiveness of different predictive models in clinical decision making, which can help decision makers weigh benefits and risks at different thresholds to choose the most appropriate decision strategy [35]. NRI was employed to evaluate the predictive performance of the three models and was rated as follows: low risk: 0%–30%, medium risk: 30%–60%, and high risk: 60%–100% (the bootstrap resampling process was repeated 100 times). After these evaluations, a dynamic nomogram was generated by selecting the optimal prediction model.

To evaluate the performance of the above optimal prediction model, two models were introduced for comparison, namely HSI and ZJU index. The HSI is a NAFLD screening tool constructed by Li et al. based on 10,724 samples [19], and when HSI is ≥ 36, it is considered an indicator of fatty liver (Eq. 1). The ZJU index is a simple NAFLD prediction model constructed by Wang et al. based on prospective research [24], and when the ZJU index > 38, it is judged as NAFLD (Eq. 2). A multi-dimensional evaluation was conducted using the following five indicators: accuracy, precision, recall, F1-score, and balanced accuracy.

1 $$HSI = 8 \times \frac{ALT}{AST} + BMI(+2, if type 2 diabetes; +2, if females)$$

2 $$ZJU = BMI + FPG + TG + 3 \times \frac{ALT}{AST}(+2, if females)$$

### Construction of a dynamic nomogram and Bayesian network model

**Fig. 1:**
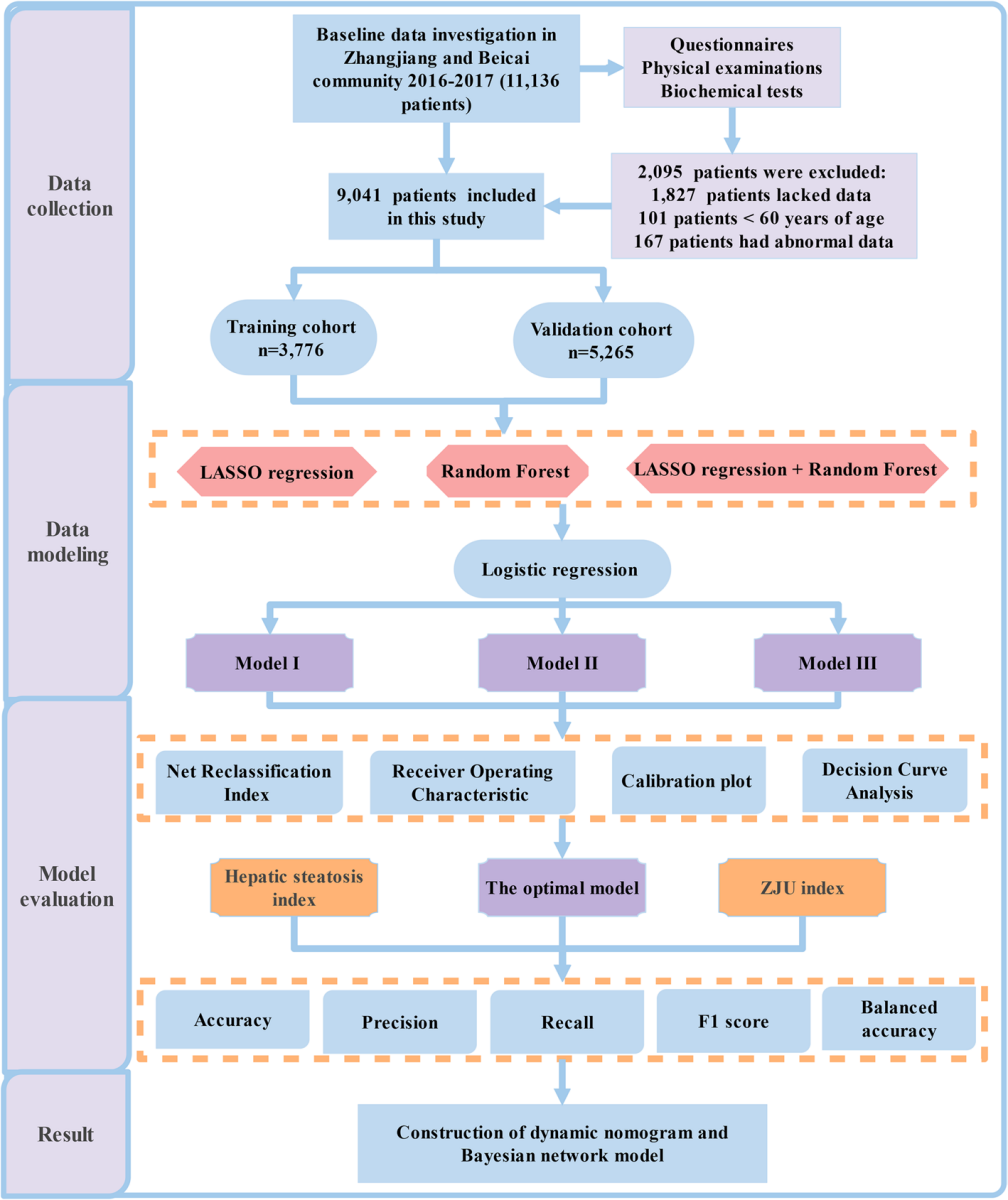
Study flowchart. LASSO, least absolute shrinkage and selection operator

The nomogram is a way to translate complex regression equations into visual graphs that makes predictive modelling results more readable and, in turn, easier to contribute to patient assessment. Based on the above comprehensive evaluation, the optimal model was constructed using the DynNom package and a corresponding online web application was developed. The Bayesian network model can use the conditional probability distribution determined using network structure and parameter learning to infer and calculate uncertain events. Netica software can intuitively display the complex network mechanism between outcomes and variables. Therefore, a naive Bayesian network model was constructed, with the optimal model and the feature variables contained in NAFLD as attribute child nodes and final parent nodes, respectively, and then imported into the training dataset for network structure and parameter learning. The specific steps of this study can be found in the flowchart shown in Fig. 1.

### Statistical analysis

The training dataset, including metrics such as physical examinations and biochemical tests, was presented as mean ± standard deviation, followed by *t*-tests. Statistical analyses were conducted using IBM SPSS Statistics software (version: 20.0; International Business Machines Corporation, Armonk, New York, U.S.), Venn (version: 2.1.0, https://bioinfogp.cnb.csic.es/tools/venny/index.html) and R statistical software (version: 4.3.1; R Foundation for Statistical Computing, Vienna, Austria land). For all analyses, *P* < 0.05 was considered statistically significant.

## Results

### Participant characteristics

The participants were categorised into the no-NAFLD (*n* = 1,787) and NAFLD (*n* = 1,989; 52.7%) groups based on the clinical diagnostic criteria [28]. The baseline characteristics of the two groups are presented in Table 1.

**Table 1:** Characteristics of the study participants (*N* = 3776)

Demographic characteristics	No-NAFLD (*n* = 1,787)	NAFLD (*n* = 1,989)	*P*-value
Gender (male/female)	858/929	868/1121	0.007
Age (years)	72.76 ± 6.65	72.18 ± 6.33	0.002
Diabetes (no/yes)	1575/212	1650/551	< 0.001
SBP (mmHg)	140.06 ± 22.28	144.89 ± 20.73	< 0.001
DBP (mmHg)	83.20 ± 12.01	85.80 ± 10.99	< 0.001
BMI (kg/m^2^)	22.51 ± 2.92	25.82 ± 2.96	< 0.001
ALB (g/L)	44.54 ± 2.74	45.06 ± 2.70	< 0.001
ALT (U/L)	15.04 ± 11.13	18.24 ± 11.58	< 0.001
AST (U/L)	19.62 ± 7.30	20.01 ± 7.46	0.113
RBC (10^12^/L)	4.43 ± 0.45	4.56 ± 0.43	< 0.001
Urea (mmol/L)	5.85 ± 1.57	5.68 ± 1.42	0.001
FPG (mmol/L)	5.46 ± 1.85	5.74 ± 1.92	< 0.001
HGB (g/L)	133.78 ± 13.71	137.60 ± 12.97	< 0.001
PLT (10^9^/L)	194.81 ± 47.30	199.51 ± 47.89	0.002
TC (mmol/L)	4.74 ± 0.92	4.89 ± 0.91	< 0.001
TB (umol/L)	11.04 ± 4.53	11.10 ± 4.55	0.653
CRE (umol/L)	76.65 ± 18.91	75.76 ± 18.41	0.142
LDL (mmol/L)	3.01 ± 0.79	3.13 ± 0.80	< 0.001
TG (mmol/L)	1.15 ± 0.72	1.61 ± 0.99	< 0.001
UA (umol/L)	290.02 ± 80.89	314.37 ± 1.87	< 0.001
AFP (ng/mL)	5.48 ± 4.62	5.35 ± 3.66	0.321
LYMPH (10^9^/L)	1.92 ± 0.60	2.10 ± 0.62	< 0.001
NEUT (10^9^/L)	3.46 ± 1.15	3.65 ± 1.13	< 0.001

*LDL* Low-density lipoprotein level, *BMI* Body mass index, *AST* Aspartate aminotransferase level, *RBC* Red blood cell count, *Urea* Urea level, *FPG* Fasting plasma glucose, *HGB* Hemoglobin, *PLT* Platelet count, *TC* Total cholesterol, *TB* Total bilirubin, *ALB* Albumin level, *CRE* Creatinine level, *TG* Triglyceride level, *UA* Uric acid level; *DBP* Diastolic blood pressure, *AFP* Alpha-fetoprotein level, *LYMPH* Lymphocyte count, *SBP* Systolic blood pressure, *NEUT* Neutrophil count, *ALT* Alanine transaminase level

### Identification of risk factors

**Fig. 2:**
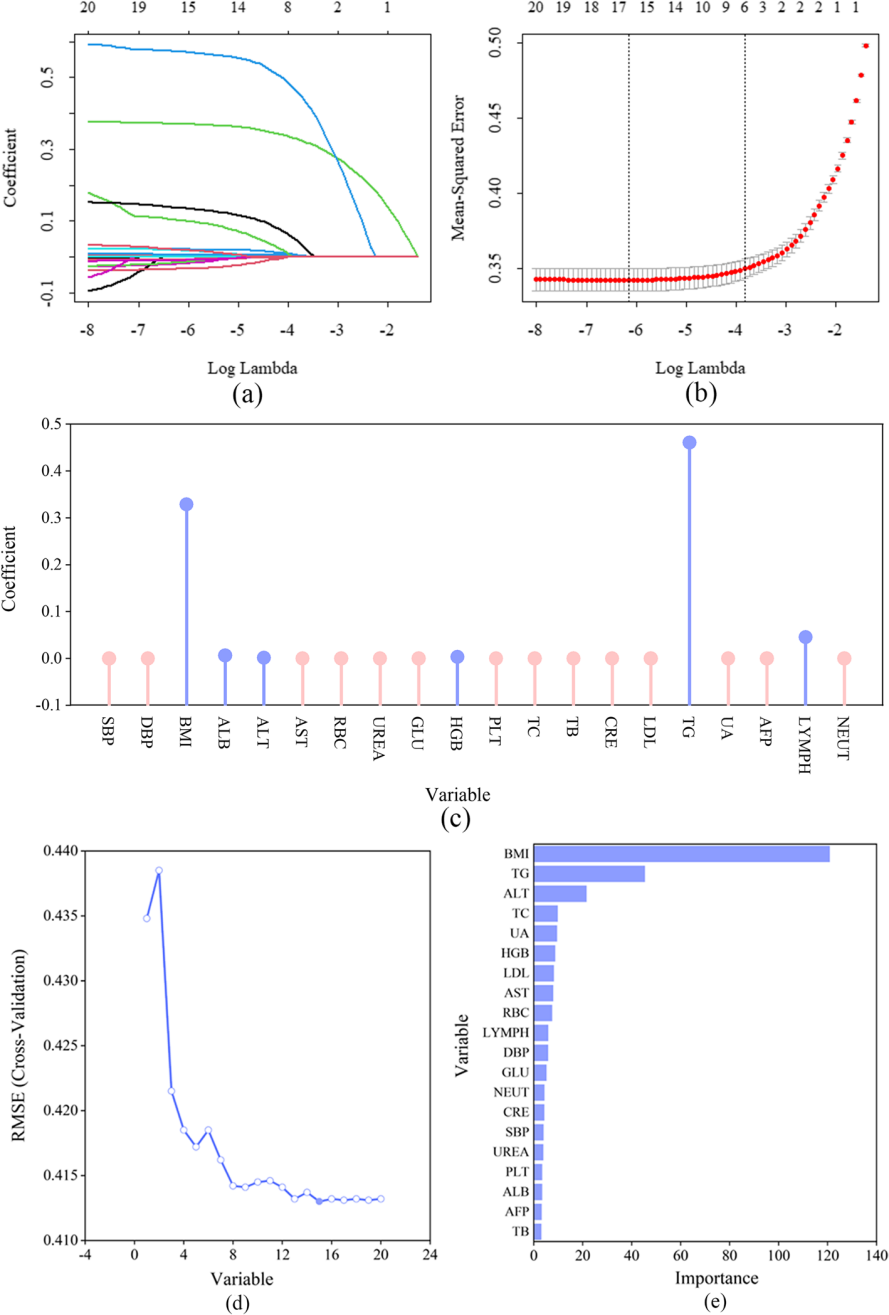
Screening of characteristic variables. **a** Six variables with non-zero coefficients were selected based on the optimal value of the parameter lambda. **b** After validating the optimal lambda value, the relationship between partial likelihood deviance and log (lambda) was plotted. The dashed vertical line represents the 1 − SE standard. **c** The orange solid point indicates that the coefficient of the variable is zero; the blue solid point indicates that the coefficient of the characteristic variable is not zero. **d** Based on the feature recursive elimination method, the RF model was used for feature extraction. Overall, 15 important variables were retained. **e** Ranking of feature importance of RF after tenfold cross-validation. RF, random forest, BMI, body mass index; TG, triglyceride level; ALT, alanine transaminase level; TC, total cholesterol; UA, uric acid level; HGB, haemoglobin; LDL, low-density lipoprotein level; AST, aspartate aminotransferase level; RBC, red blood cell count; LYMPH, lymphocyte count; DBP, diastolic blood pressure; GLU,; NEUT, neutrophil count; CRE, creatinine level; SBP, systolic blood pressure; PLT, platelet count; ALB, albumin level; AFP, alpha-fetoprotein level; TB, total bilirubin

Using the LASSO regression model to screen variables and conducting tenfold cross-validation, six feature variables (body mass index [BMI], albumin level [ALB], ALT, haemoglobin [HGB], TG, and lymphocyte count [LYMPH]) with non-zero coefficients were ultimately selected, and the optimal lambda coefficient was 0.000331. Based on RF analysis results, when the number of variables was 15 (BMI, TG, ALT, uric acid level [UA], red blood cell count, low-density lipoprotein level, total cholesterol, HGB, LYMPH, aspartate aminotransferase level [AST], DBP [diastolic blood pressure], creatinine level [CRE], SBP [systolic blood pressure], fasting plasma glucose, and neutrophil count), the model had the highest fitting accuracy, with a root mean square error of 0.413, as shown in Fig. 2.

### Construction of the prediction model

Based on the results of LASSO regression analysis, with six non-zero coefficient variables and the NAFLD incidence rate as independent and dependent variables, respectively (see Additional file 1), the following equation for Model I was obtained:

3 $${NAFLD}_{Model I} = -13.075 + 0.381 \times BMI + 0.032 \times ALB + 0.010 \times ALT + 0.009 \times HGB + 0.641 \times TG + 0.177 \times LYMPH$$

Based on the RF analysis results, BMI, ALT, TG, UA, CRE, AST, and LYMPH were identified as important independent predictors of NAFLD among the 15 retained features and were used as independent variables to construct Model II (see Additional file 2). The following equation was obtained:

4 $${NAFLD}_{Model II} = -9.752 + 0.376 \times BMI + 0.028 \times ALT + 0.621 \times TG + 0.001 \times UA - 0.009 \times CRE - 0.031 \times AST + 0.174 \times LYMPH$$

**Fig. 3:**
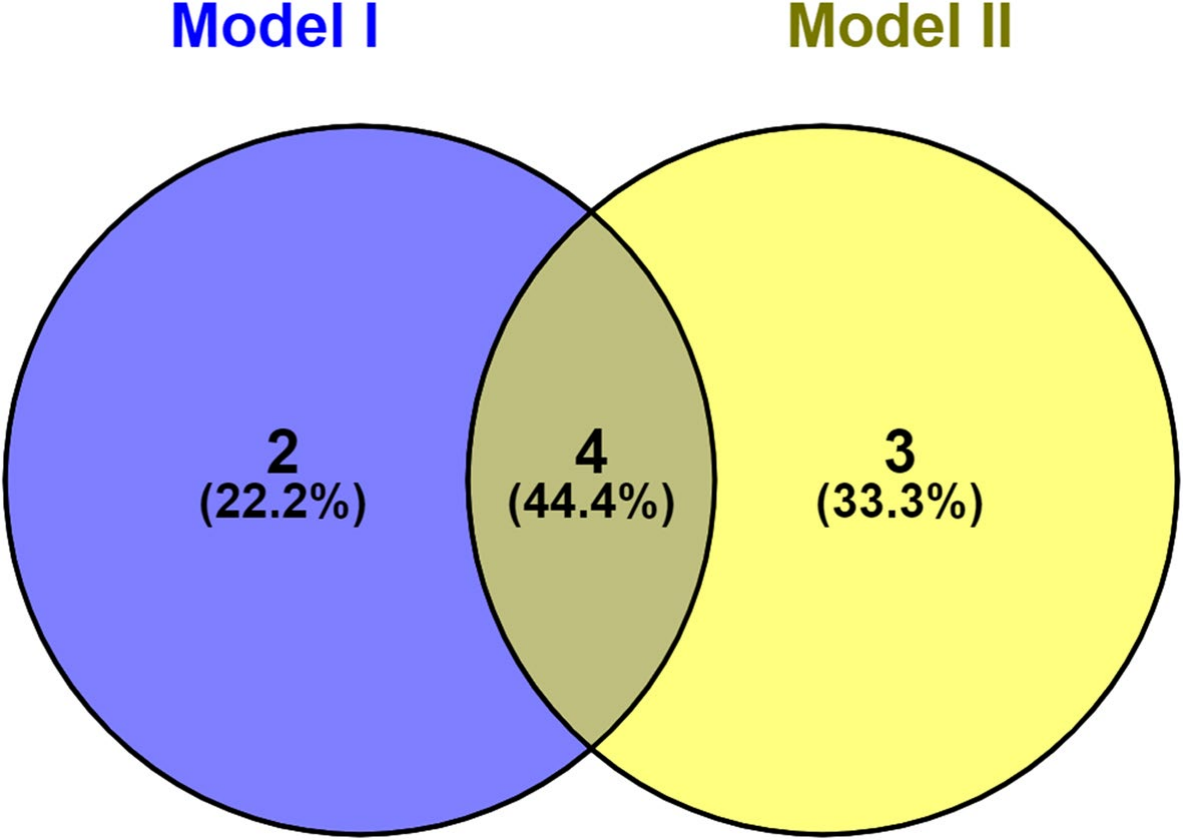
Screening of variables in Model III. Selection of the intersection of the variables in Models I and II. These four intersecting variables were used to construct Model III

Intersecting variables in Models I and II (Fig. 3) were included as independent variables to construct Model III (see Additional file 3). The following equation was generated:

5 $${NAFLD}_{Model III} = -10.610 + 0.383 \times BMI + 0.012 \times ALT + 0.673 \times TG + 0.194 \times LYMPH$$

### Evaluation of the prediction model

**Fig. 4:**
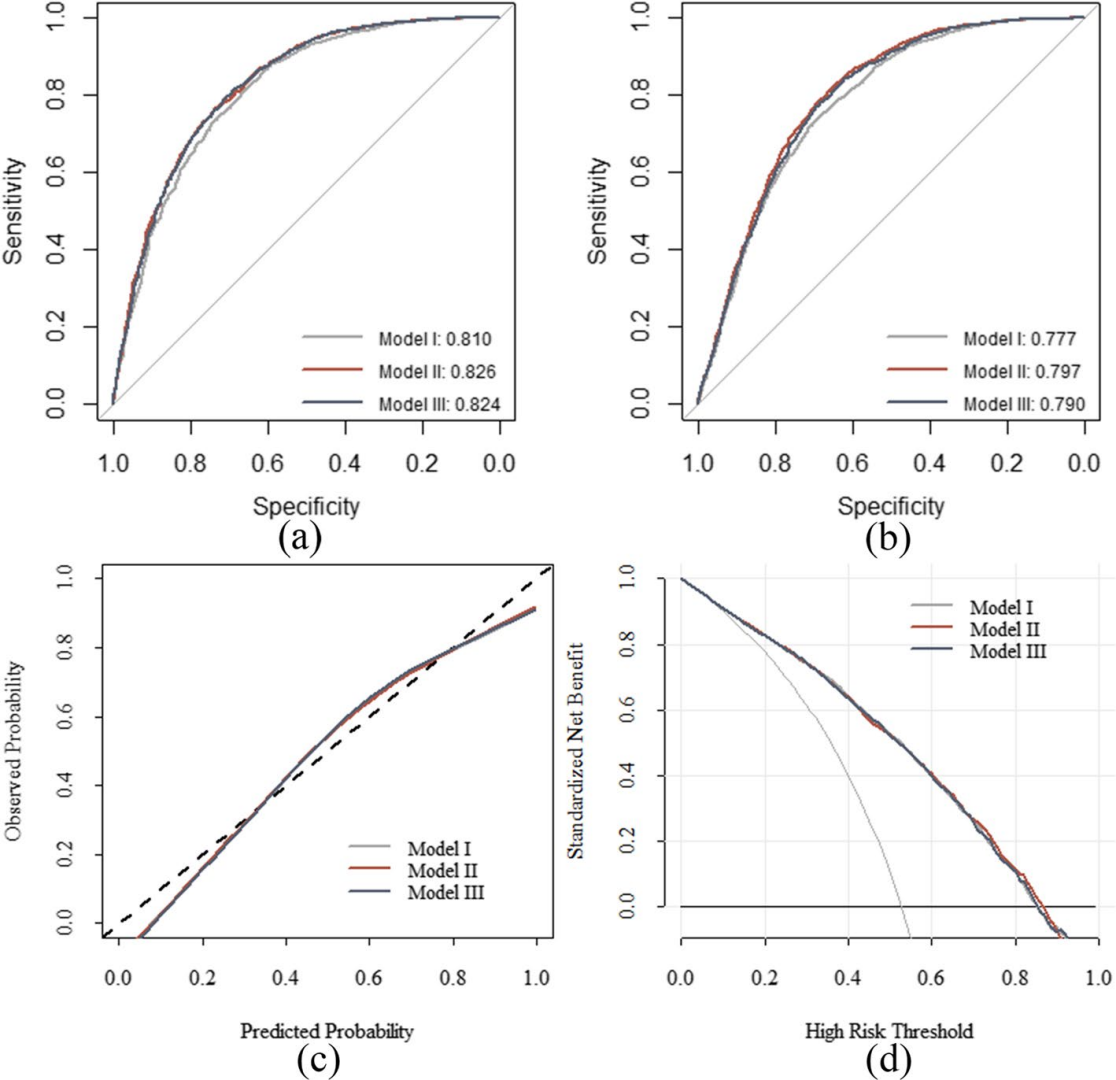
Evaluation of three models. Analyses of the ROC curves of the three non-alcoholic fatty liver disease prediction models for the training (**a**) and validation datasets (**b**) are shown. The x and y axes represent specificity and sensitivity, respectively. **c** Calibration plots of the risk prediction models from the training dataset. The diagonal dashed line denotes the perfect prediction of an ideal model, whereas the solid line denotes the model’s performance. (**d**) Decision curve analysis for the risk prediction models. The black solid line denotes the net benefit when all participants were negative and were not treated, whereas the grey solid line denotes the net benefit when all participants were positive and received treatment. The further the decision curve is from the black and grey solid lines, the more useful the risk prediction model is in clinical practice. ROC, receiver operating characteristic

**Fig. 5:**
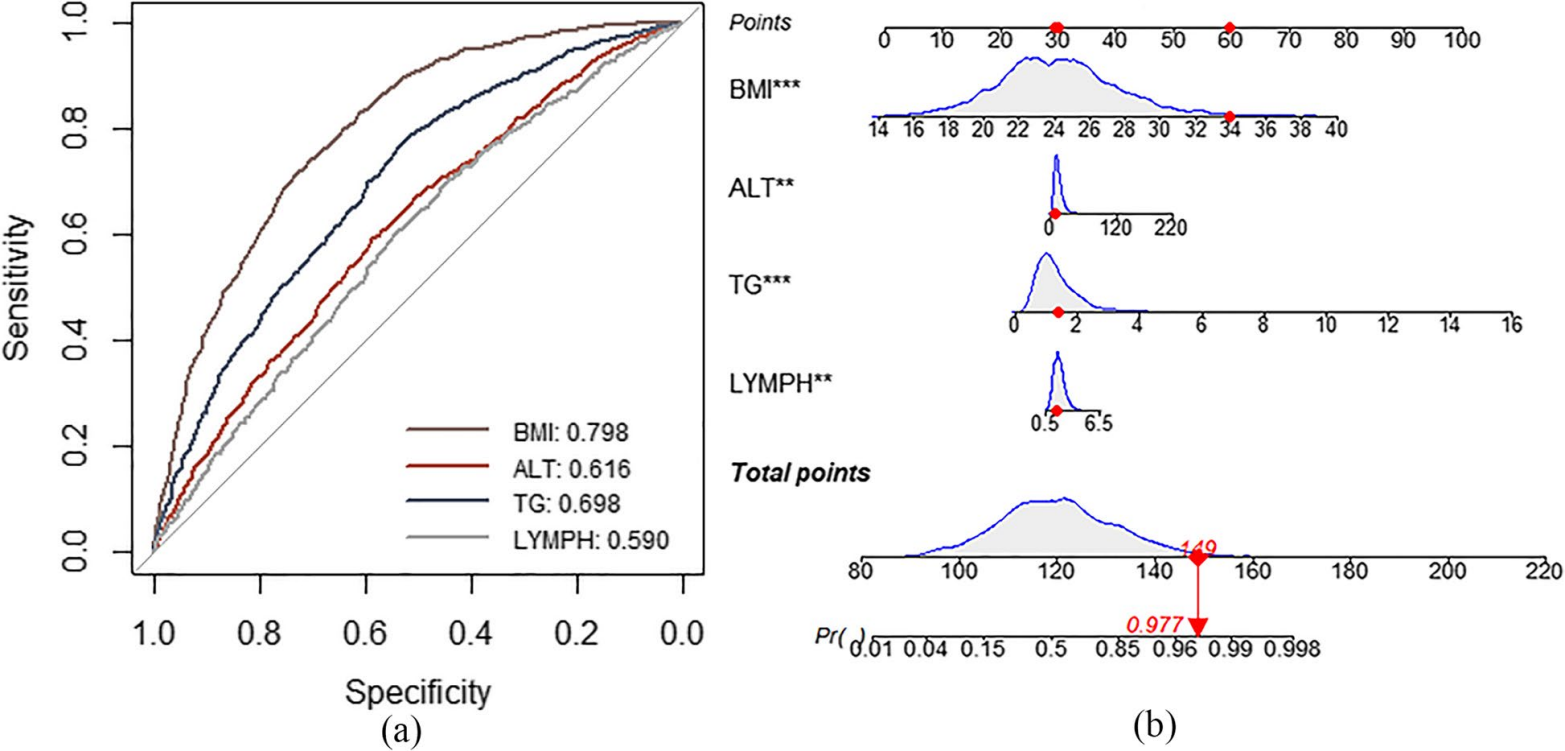
The receiver operating characteristic curves for each variable in Model III and proposed dynamic nomogram. **a** The ROC curves for each variable in Model III are shown for the training dataset. The x-axis represents the false positive rate predicted using the model, and the y-axis represents the true positive rate predicted using the model. **b** A dynamic nomogram was created based on Model III to predict an individual’s risk of developing NAFLD. Based on a patient’s lymphocyte count (1.83 × 10^9^/L), alanine transaminase level (8.9 U/L), triglyceride level (1.39 mmol/L), and body mass index (33.9 kg/m^2^), the predicted probability of the development of NAFLD was 0.977, indicating that this patient has a 97.7% chance of having NAFLD. BMI, body mass index; ALT, alanine transaminase level; TG, triglyceride level; LYMPH, lymphocyte count; NAFLD, non-alcoholic fatty liver disease; ROC, receiver operating characteristic

The AUC values for Models I, II, and III were 0.810 (95% CI: 0.796–0.824), 0.826 (95% CI: 0.812–0.839), and 0.825 (95% CI: 0.811–0.837), respectively, for the training dataset and 0.777 (95% CI: 0.765–0.790), 0.797 (95% CI: 0.784–0.809), and 0.790 (95% CI: 0.777–0.802), respectively, for the validation dataset, as shown in Fig. 4a and b. The prediction accuracies of Models II and III were higher than those of Model I but were not significantly different from each other (Table 2). The three prediction models were moderately consistent, as shown in Fig. 4c. No significant differences were found between the DCA curves of the three models. The DCA curves indicated a threshold probability of 20%–80% for the benefits of predicting NAFLD risk using the prediction model versus implementing interventions in the general population, as shown in Fig. 4d. The risk prediction capabilities of the three models were not significantly different (*P* = 0.275; Table 3). Because Model III could achieve high prediction performance with fewer indicators, it was considered the optimal prediction model. This model mainly included the following four indicators: BMI, ALT, TG, and LYMPH. The AUC values for BMI, ALT, TG, and LYMPH were 0.798, 0.616, 0.689, and 0.590, respectively, as shown in Fig. 5a. To evaluate the effectiveness of Model III, the HSI and ZJU index were introduced to compare accuracy, precision, recall, F1-score, and balanced accuracy in five aspects. The findings indicate that the model established in this study outperformed HSI and ZJU in both training and testing datasets, as shown in Tables 4 and 5.

**Table 2:** Pairwise comparisons of the receiver operating characteristic curves of the three models

Statistics	Model I-II	Model I-III	Model II-III
*Z*	-5.808	-5.565	1.472
*P*	< 0.001	< 0.001	0.141

**Table 3:** Predictive abilities of the three models

Models	NRI	*P*-value	2.5% CI	97.5% CI
Model I ~ II	0.013	0.162	-0.005	0.032
Model I ~ III	0.004	0.603	-0.012	0.021
Model II ~ III	-0.009	0.275	-0.025	0.007

*NRI* Net reclassification index, *CI* Confidence interval

**Table 4:** Different metrics to evaluate the performance of NAFLD risk prediction models in training data

Model	Accuracy	Precision	Recall	F1-score	Balanced accuracy
Model III	0.716	0.808	0.605	0.692	0.7226
HSI	0.610	0.827	0.328	0.470	0.6258
ZJU	0.616	0.810	0.354	0.493	0.6311

**Table 5:** Different metrics to evaluate the performance of NAFLD risk prediction models in test data

Model	Accuracy	Precision	Recall	F1-score	Balanced accuracy
Model III	0.678	0.783	0.558	0.652	0.687
HSI	0.629	0.783	0.430	0.555	0.645
ZJU	0.633	0.790	0.432	0.559	0.649

**Fig. 6:**
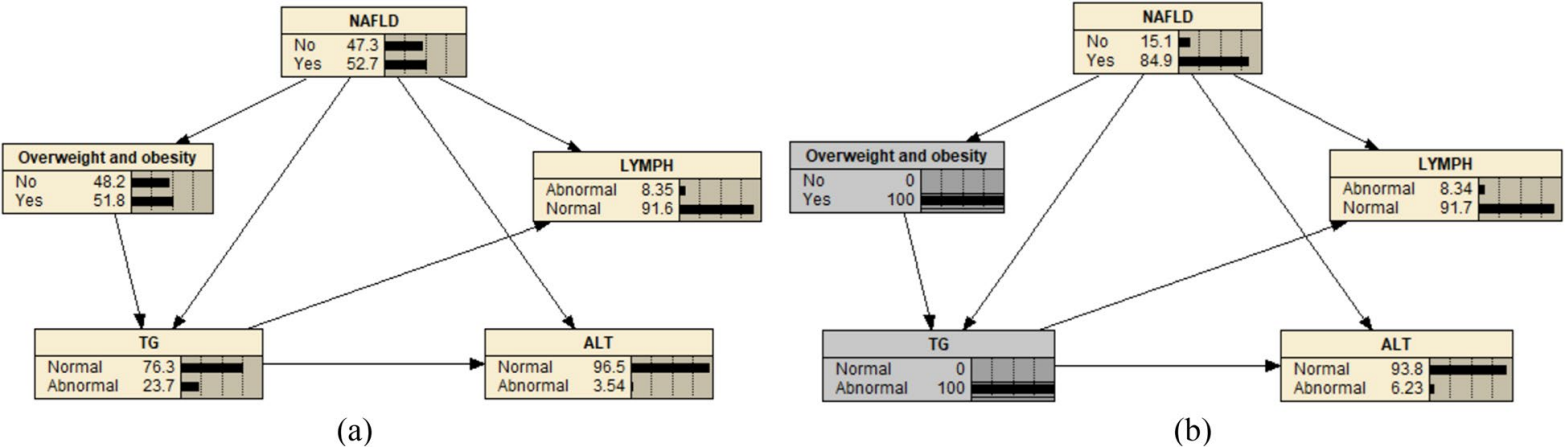
Bayesian network model for predicting the risk of NAFLD in older adults. **a** A Bayesian network model was constructed to predict the risk of NAFLD in older adults based on the four variables of Model III. **b** When a 65-year-old individual is overweight and has abnormal TG indicators, the risk of having NAFLD is 84.9%.NAFLD, non-alcoholic fatty liver disease; TG, triglyceride; LYMPH, lymphocyte count; ALT, alanine transaminase level

Meanwhile, this study developed a dynamic nomogram to calculate and visualise the risk of NAFLD in Chinese older adults, as shown in Fig. 5b. This study established a dynamic nomogram online application to predict the NAFLD incidence risk for older Chinese adults; the URL is as follows: https://doctorpan.shinyapps.io/NAFLDapp. Additionally, this study used the TAN structure learning function of Netica software, with NAFLD and four variables in Model III as the parent and attribute nodes, respectively. We imported the training set to learn the structure and parameters of the TAN Bayesian network, and the established network is shown in Fig. 6a. Assuming that a certain research participant is known to be 65 years old, overweight, and has abnormal TG levels, if this information is inputted into the Bayesian network structure constructed using Netica software, the estimated risk of NAFLD is 84.9%, as shown in Fig. 6b. Therefore, it can be found that using Bayesian networks for risk prediction is not only easy to operate but also has good visibility. Risk prediction can still be conducted without knowing all patients’ health information. For example, the unknown weight, ALT, and other conditions of the research participants in the above example can also be used to determine the risk of outcome occurrence. This is crucial for solving the problem of a large amount of missing information from the research participants in practical work.

## Discussion

With the rise in economic status, individuals' dietary habits and lifestyles have undergone gradual alterations, resulting in a yearly surge in the occurrence of NAFLD [36]. Due to the widespread availability of the hepatitis B vaccine, the chronic noncommunicable disease, NAFLD, is gradually emerging as the primary liver disease worldwide. NAFLD has long been a significant and unavoidable challenge to the healthcare system; therefore, early detection, diagnosis, and preventive measures are critical to managing people with NAFLD. Currently, most people with NAFLD are diagnosed incidentally during imaging tests for a common annual physical examination or other medical conditions. Because abdominal ultrasound is not routinely performed, community physician suspicion of NAFLD is critical during routine physical examinations. In contrast to imaging techniques, BMI, ALT, TG, and LYMPH can be routinely monitored on a routine basis during the annual health check-up.

Therefore, the development of a simple and practical diagnostic tool is essential to solve this problem. Through the analysis of physical examination indicators, this study developed and validated a dynamic nomogram and Bayesian network model to predict NAFLD risk in older Chinese adults using four easily collected features (BMI, ALT, TG, and LYMPH). In this study, Model III, constructed by combining the common variables screened using the LASSO regression and RF models, has higher predictive power (AUC = 0.824) and contains fewer variables than Models I and II, constructed using the LASSO regression analysis and RF algorithm alone. Simultaneously, compared with the existing NAFLD prediction models (HSI and ZJU) developed for Asian populations, Model III we constructed is more suitable for older adults aged ≥ 60 years in China. HSI originated from a study involving more than 10,000 participants in South Korea with limited external utility; ZJU is a NAFLD prediction model developed for the entire Chinese population, with less targeted to special populations (elderly groups). Additionally, we built a Bayesian network model based on key variables that is simple to operate, has good visibility, and can predict risks without all patients’ health information, such as the ALT, LYMPH, and other situations of unknown research participants in the above examples; this model can also determine the risk of occurrence of outcomes, which is crucial to solving the problem of a large amount of missing information in research participants in real-world studies.

### Major risk factors for NAFLD in older adults

BMI has been consistently identified as a factor in the development of NAFLD. Hepatocytes have been attributed with an adipocyte-like function when there is a reduction in adipose tissue's ability to store surplus energy [37]. In typical cases of obesity, hepatocytes store excess lipids, primarily as TGs, resulting in simple steatosis [38]. The increased lipolysis and decreased fatty acid uptake in subcutaneous adipose tissue leads to raised levels of circulating free fatty acids, which may lead to an accumulation of fat in the liver, and subsequently, IR [39]. Insulin-resistant adipocytes secrete free fatty acids (FFAs), and elevated levels of FFAs in the liver lead to increased lipid synthesis and gluconeogenesis [40, 41]. The inability of liver cells to process excess FFAs leads to fat cell apoptosis, which is an essential feature of NASH [42]. Being overweight or obese has been identified as a risk factor for NAFLD [3, 43–46]. For instance, a prospective study carried out in Cuba has revealed a clear link between weight loss at 52 weeks and the reduction of non-alcoholic steatohepatitis symptoms in a dose-dependent manner [47]. Furthermore, Liu et al. established different NAFLD models using seven machine learning methods and found that high BMI level was the strongest predictor of risk, which aligns with the findings of this study [21].

ALT is a predictive factor for NAFLD, recent studies have shown [48, 49]. ALT is a vital enzyme in the human body, primarily located in liver cells and, its clearance in the plasma is conducted by hepatocytes, with no discernible contribution from sinusoidal cells [50]. Therefore, elevated ALT serum levels are generally regarded as an indicator of liver impairment [51]. In addition, elevated ALT levels indicate impaired insulin signalling, which can lead to hepatic IR, which plays a key role in the pathogenesis of NAFLD [38, 48]. In China, a 4-year cohort study of 13,240 participants with NAFLD found that participants who eventually developed NAFLD had significant differences in ALT levels from those who did not develop the disease [52]. It was found in a meta-analysis that one-quarter of the NAFLD patients have normal ALT levels [53]. In this study, ALT was also selected in the bootstrap samples of all three models.

Changes in TG levels may be a predictor of NAFLD risk [54]. A simple algorithm for predicting the risk of fatty liver developed based on public database identified TG as a common predictive variable [20]. Elevated TG levels lead to increased FFAs during lipolysis, subsequently resulting in worsened insulin sensitivity. The activation of the body's oxidative stress responses may lead to IR in tissues [55]. IR can, in turn, stimulate hepatic lipid synthesis by inducing TG hydrolysis and the breakdown of newly formed fat in adipose tissues [56]. As a result of this circulation, lipids accumulate in liver cells [57]. A study conducted on Malaysian children highlights that individuals diagnosed with NAFLD have noticeably elevated TG levels in comparison to non-NAFLD patients [58].

Dysregulation of lipid metabolism in NAFLD leads to intrahepatic CD4 + T lymphocyte loss selectively, implying that NAFLD patients have lower LYMPH profiles than those without NAFLD [59]. As a vital hemopoietic organ during human embryonic development [60], the degree of fatty liver disease is correlated with various blood biochemical markers. Elevated biochemical markers reflect the condition of the liver, and LYMPH can be routinely used to determine the progression of fatty liver disease [61]. From Fig. 2 and Table 1, it can be seen that the lymphocyte count of NAFLD patients increased, and the main role of these blood cells is evident in the risk prediction model of NAFLD. However, it is interesting to note the findings of increased lymphocyte counts and obesity in previous studies, suggesting that weight changes may cause immunological changes at the peripheral level [62]. Another study also suggests that increased lymphocytes can also cause weight gain [63], indicating that obesity and lymphocytes may have an interaction. Is leptin concentration in obese individuals a mediator associated with lymphocytes now? As in a mouse controlled experiment, researchers found that leptin has a direct impact on T cell-mediated immunity [64]. Alternatively, the viral etiology may provide another explanation for the link between lymphocytes and obesity, such as adenoviruses activating adipogenesis in adipose tissue to cause obesity, activating pro-inflammatory pathways that affect lymphocyte levels in the body and thus affect the occurrence of NAFLD [65]. MCP-1 is the major lymphocyte chemoattractant, and Carr et al. found that lymphocytes have a dose-dependent and chemotactic response to MCP-1 that can be replicated [66]. In addition, MCP-1 plays a crucial role in adenovirus 36 induced obesity, as shown in a classic study that adenovirus 36 serves as an agent for obesity maintenance by inducing inflammation and increasing MCP-1 [67].

### Health management strategies for older Chinese adults

With the increase in people’s age, the incidence rate of diseases will also increase significantly. Regular physical examinations can effectively help people monitor their health status, identify health risks as soon as possible, and are crucial for health management. They can also be considered the basic pillars of health management. Community health service medical personnel can use our developed model to input indicator data (BMI, ALT, TG, and LYMPH) acquired from physical examinations to obtain individual-specific NAFLD disease risk. Aiming at the elderly population with a low risk of NAFLD, health education methods is mainly used to impart certain nutrition and exercise knowledge to help them better understand dietary nutrition collocation in daily life and choose appropriate exercise methods. Additionally, medical staff can use regular physical examinations to timely understand their physiological status through health education, urged them to maintain a healthy lifestyle, and used regular physical examinations to track and detect their health status.

Correcting poor diet and exercise habits remains the cornerstone of preventing and treating NAFLD in middle-aged and high-risk elderly populations and patients with NAFLD. The use of diet exercise prescriptions for systematic health management mainly enables them to master certain nutritional knowledge, correctly choose low-fat, high-protein, low-calorie, high-fibre foods, control calorie intake, and change their eating habits. Diets with a low glycaemic index and load can reduce the hepatic lipid content and serum ALT level in patients with NAFLD [68]. Diets low in fat (saturated fatty acid intake < 10%) and carbohydrates (< 50% of total kcal) can effectively lower serum ALT levels [69–71]. Exercise therapy can promote fat metabolism and increase energy consumption, and it can be combined with dietary therapy to achieve slow weight loss and prevent further aggravation of liver damage caused by excessive weight loss. For every kilogramme of weight lost, the ALT level decreases by 0.83 units [72], and the TG level decreases by approximately 8 mg/dL [73]. Aerobic exercise can also reduce the TG level by 10%–20% [73].

In addition to improving lifestyle, some studies have demonstrated that NAFLD can be treated by regulating intestinal flora, such as by adding probiotics, prebiotics, and symbiotic supplements [74, 75]. The combination of probiotics and *Salvia miltiorrhiza* polysaccharide can regulate intestinal flora and improve IR, thereby alleviating the symptoms of NAFLD [76]. Liu et al. conducted a meta-analysis of 15 clinically randomised controlled trials and found that supplementing probiotics and synthetic bacteria in NAFLD patients could significantly improve fatty liver degeneration and TG levels and reduce ALT levels, among others [77]. Currently, limited studies exist on the probiotic adjuvant treatment of NAFLD, and the sample sizes are low. Clinical research in this area is still in the initial stages [78]. In the future, the treatment and management of liver diseases may benefit from the application of probiotics in the future, which is considered a new treatment method. Currently, the strategies used to control NAFLD and its progression include changing lifestyles (nutritious diets, aerobic exercise, and resistance training) and using hypoglycaemic drugs and antioxidants, among others [11, 79]. However, lifestyle changes focusing on weight loss remain the primary method for preventing and controlling disease development [11, 14, 69]. Community health care providers can use this NAFLD risk prediction model to assess the risk of NAFLD in older adults, propose health management recommendations based on diet and exercise for individuals at risk, and conduct personalised interventions, which can reduce the prevalence of NAFLD among older adults, improve the quality of life of the older adults, and reduce the family economic stress and social health system pressure.

### Strength and study limitation

The main advantage of this study is to construct a NAFLD risk prediction model for the elderly population in China with fewer indicators and higher accuracy, based on the widely used HSI and ZJU index in Chinese adults. And for the first time, lymphocytes were also found to be one of the risk factors for predicting NAFLD, which will help improve the performance of the prediction model. However, this study did not analyze potential strong predictive factors associated with NAFLD, such as gamma glutamyl transpeptidase, alkaline phosphatases, direct bilirubin, waist circumference and HDL. Therefore, in future research, there is a need to continue analysing other potential risk factors to enrich the NAFLD prediction model. In addition, the dynamic nomogram proposed in this study requires external validation in a larger population.

## Conclusions

This study developed a simple and effective dynamic nomogram that healthcare providers can use to determine the risk of NAFLD in older adults. The model constructed in this study has stronger specificity and higher predictive ability than the existing models. Based on the results of this study, lifestyle modifications (including healthy diets and exercise) should be implemented for older adults who are susceptible to NAFLD. Healthcare providers can use this dynamic nomogram to monitor the risk of NAFLD and personalise health interventions to improve the individual quality of life and reduce the economic and healthcare burdens of NAFLD in older adults, their families, and society.

### Supplementary Information

**Additional file 1: Table 1.** Risk factors included in Model I.

**Additional file 2: Table 2.** Risk factors included in Model II.

**Additional file 3: Table 3.** Risk factors included in Model III.

## Data Availability

The raw data used in this article will be available to the corresponding authors upon request.

## Abbreviations

AFP: Alpha-fetoprotein level
ALT: Alanine aminotransferase
AST: Aspartate aminotransferase level
AUC: Area under the ROC curve
BMI: Body mass index
CI: Confidence intervals
CRE: Creatinine level
DBP: Diastolic blood pressure
DCA: Decision curve analysis
FFA: Free fatty acids
HGB: Haemoglobin
HIS: Hepatic steatosis index
IR: Insulin resistance
LASSO: Least absolute shrinkage and selection operator
LYMPH: Lymphocyte count
NAFLD: Non-alcoholic fatty liver disease
NRI: Net reclassification index
OR: Odds ratio
PLT: Platelet count
IQR: Interquartile range
RF: Random forest
ROC: Receiver operating characteristic
SBP: Systolic blood pressure
TG: Triglyceride
