# Supplementary material for: Non-alcoholic fatty liver disease risk prediction model and health management strategies for older Chinese adults: a cross-sectional study
Source: Lipids Health Dis. 2023 Nov 25;22:205. doi: 10.1186/s12944-023-01966-1 (PMC10675849; doi:10.1186/s12944-023-01966-1)
Supplement: Supplementary file 1 — Additional file 1: Table 1. Risk factors included in Model I. [file 12944_2023_1966_MOESM1_ESM.docx]

**Table 1** Risk factors included in Model I

| Intercept and variable | Model I | | | | | |
| --- | --- | --- | --- | --- | --- | --- |
|  | β | z-value | P | OR | 2.5% CI | 97.5% CI |
| Intercept | -13.075 | -16.193 | <0.001 | 2.097e-06 | 4.241e-07 | 1.006e-05 |
| BMI | 0.381 | 24.015 | <0.001 | 1.464 | 1.420 | 1.511 |
| ALB | 0.032 | 2.139 | 0.032 | 1.033 | 1.003 | 1.063 |
| ALT | 0.010 | 2.471 | 0.013 | 1.010 | 1.002 | 1.018 |
| HGB | 0.009 | 2.833 | 0.005 | 1.009 | 1.003 | 1.015 |
| TG | 0.641 | 10.337 | <0.001 | 1.898 | 1.684 | 2.147 |
| LYMPH | 0.177 | 2.740 | 0.006 | 1.193 | 1.052 | 1.355 |

OR: odds ratio; CI: confidence interval; BMI: body mass index; ALB: albumin level; ALT: alanine transaminase level; HGB: hemoglobin; TG: triglyceride level; LYMPH: lymphocyte count.
