# Supplementary material for: Non-alcoholic fatty liver disease risk prediction model and health management strategies for older Chinese adults: a cross-sectional study
Source: Lipids Health Dis. 2023 Nov 25;22:205. doi: 10.1186/s12944-023-01966-1 (PMC10675849; doi:10.1186/s12944-023-01966-1)
Supplement: Supplementary file 2 — Additional file 2: Table 2. Risk factors included in Model II. [file 12944_2023_1966_MOESM2_ESM.docx]

**Table 2** Risk factors included in Model II

| Intercept and variable | Model II | | | | | |
| --- | --- | --- | --- | --- | --- | --- |
|  | β | *z*-value | *P* | OR | 2.5% CI | 97.5% CI |
| Intercept | -9.752 | -21.941 | <0.001 | 5.815e-05 | 2.406e-05 | 1.375e-04 |
| BMI | 0.376 | 23.428 | <0.001 | 1.456 | 1.412 | 1.503 |
| ALT | 0.028 | 4.257 | <0.001 | 1.028 | 1.015 | 1.041 |
| TG | 0.621 | 9.968 | <0.001 | 1.860 | 1.650 | 2.106 |
| UA | 0.001 | 2.460 | 0.014 | 1.001 | 1.000 | 1.003 |
| CRE | -0.009 | -3.423 | 0.001 | 0.991 | 0.986 | 0.996 |
| AST | -0.031 | -3.293 | 0.001 | 0.970 | 0.952 | 0.987 |
| LYMPH | 0.174 | 2.690 | 0.007 | 1.190 | 1.048 | 1.350 |

OR: odds ratio; CI: confidence interval; BMI: body mass index; ALT: alanine transaminase level; TG: triglyceride level; UA: uric acid level; CRE: creatinine level; AST: aspartate aminotransferase level; LYMPH: lymphocyte count.
