# Supplementary material for: Non-alcoholic fatty liver disease risk prediction model and health management strategies for older Chinese adults: a cross-sectional study
Source: Lipids Health Dis. 2023 Nov 25;22:205. doi: 10.1186/s12944-023-01966-1 (PMC10675849; doi:10.1186/s12944-023-01966-1)
Supplement: Supplementary file 3 — Additional file 3: Table 3. Risk factors included in Model III. [file 12944_2023_1966_MOESM3_ESM.docx]

**Table 3** Risk factors included in Model III

| Intercept and variable | Model III | | | | | |
| --- | --- | --- | --- | --- | --- | --- |
|  | β | *z*-value | *P* | OR | 2.5% CI | 97.5% CI |
| Intercept | -10.610 | -26.558 | <0.001 | 2.467e-05 | 1.114e-05 | 5.334e-05 |
| BMI | 0.383 | 24.216 | <0.001 | 1.446 | 1.442 | 1.513 |
| ALT | 0.012 | 3.079 | 0.002 | 1.012 | 1.005 | 1.020 |
| TG | 0.673 | 11.006 | <0.001 | 1.960 | 1.742 | 2.214 |
| LYMPH | 0.194 | 3.016 | 0.003 | 1.214 | 1.070 | 1.377 |

OR: odds ratio; CI: confidence interval; BMI: body mass index; ALT: alanine transaminase level; TG: triglyceride level; LYMPH: lymphocyte count.
